# Prospective analysis of intoxicated patients developing shock and/or organ failure

**DOI:** 10.55730/1300-0144.5508

**Published:** 2022-08-04

**Authors:** Faysal TEKİN, Ayça AÇIKALIN AKPINAR, Ömer TAŞKIN, Ufuk AKDAY, Nezihat Rana DİŞEL, Emre KARAKOÇ

**Affiliations:** 1 Department of Emergency Medicine, Faculty of Medicine, Çukurova University, Adana, Turkey; 2 Department of Internal Medicine, Faculty of Medicine, Çukurova University, Adana, Turkey

**Keywords:** Pop scale, hypotension, lactate, poisoning

## Abstract

**Background/aim:**

Data on intoxication patients with shock and acute organ failure is valuable for the prediction of the tailored scale prognosis of the patients in the emergency department.

**Materials and methods:**

Our study was designed prospectively as a cross-sectional and observational over the course of four years. The patients over 18 years of age were included and the epidemiological data related to development of shock and acute organ failure, the treatments, and the outcome were recorded. The organic phosphate severity score was also calculated for all patients.

**Results:**

A total of 89 patients with shock and/or acute organ failure 72 (80.9%) of the patients were males. Methanol (51 patients, 57.3%) was the most common cause of intoxications followed by cardiovascular agents. Thirty (33.7%) patients died despite all treatments and mortality was found to be higher in patients with hypotension (p = 0.031) at the time of admission to the emergency department and in those with a high organic phosphate poisoning severity index (p = 0.001). High levels of WBC, creatine, lactate, base excess and low bicarbonate and blood pH were associated with mortality. The discharge rates of patients who received extracorporeal treatment were statistically higher than those who did not receive this treatment (p = 0.001).

**Conclusion:**

The organic phosphate poisoning severity score can partially help us to predict the prognosis in all poisoning patients at the time of the first presentation. Emergency physicians may consider the development of hypotension, high creatine, lactate, base excess, low bicarbonate, and blood pH to be associated with a poor prognosis in the first hours of admission in acute poisoning patients. In these patients, it was predicted that the addition of selected extracorporeal methods without delay, in addition to the treatments that should be applied, may increase the survival rate.

## 1. Introduction

Acute poisoning is a preventable health problem that can cause deaths in adults all over the world. Approximately 1%–4% of the patients evaluated in emergency departments (ED) are intoxication patients [1]. The number of patients who apply to the ED with poisoning agents with high morbidity and mortality is increasing day by day. Epidemiological characteristics of acute poisoning patients differ from country to country. Types and routes of exposures, and the agents exposed in suicidal poisonings are closely related to the sociocultural conditions and socioeconomic status of the society.

Common acute poisoning agents in Turkey are drugs (analgesic, antidepressant, antihistamine, antihypertensive, antiepileptic, etc.), pesticides, insecticides (organophosphate, carbamate, pyrethrin group, etc.), household chemicals (bleach, sink opener, limescale removers, detergents, naphthalene, etc.), poisonous gases (carbon monoxide, suffocating gases), other chemicals, and poisonous animal bites and stings such as scorpions and snakes [2,3]. Although the National Poison Information Center provides support for consultation and obtaining antidotes in Turkey, annual statistical data related to morbidity and mortality in acute poisonings unfortunately do not accurately reflect the current situation accurately.

When studies on patients requiring critical care due to acute poisoning in different countries are examined, it is seen that some agents such as methanol and aluminum phosphide are still quite mortal [4–7]. Critical care scoring systems and poisoning severity score can be used to predict the prognosis in these patients [8]. However, these scorings are quite long and complex. There is a need for a practical and easily calculable scoring that can be evaluated at the time of admission to the ED. In acute poisoning patients with high mortality and morbidity rate, such as methanol, aluminum phosphide and paraquat, it is almost impossible to conduct studies about treatment. The aim of this study was to examine poisoning patients with high mortality rates such as those with shock and organ failure prospectively, in order to provide valuable data on diagnosis, treatment, clinical course and results to the literature. At the same time, we aimed to obtain parameters in order to create an easily calculable scoring system.

## 2. Materials and methods

Our study was conducted at Çukurova University Hospital Emergency Department between September 2015 and January 2019, after approval by the local ethics committee.

The following inclusion criteria were utilized:

- Patients over 18 years old who agreed to participate in the study whose drug or substance overdose, environmental poisoning due to sting/bite or exposure were confirmed by anamnesis, physical examination, and/or laboratory tests were included in the study if they had circulatory shock and/or single or multiple organ failure (such as acute kidney injury, acute hepatic failure, respiratory failure etc.) on admission or duration in the emergency department.
- The patients whose systolic arterial blood pressure was below 90 mmHg with a lack of adequate perfusion such as urinary output <0.5 mL/kg/h, serum lactate levels above 2 mmol/Lt, capillary refill time >2 s, or loss of consciousness and systolic blood pressure decreased by more than 40 mmHg between two consecutive measurements were all defined as shock.
- Acute kidney injury is defined as the patients have creatinine levels above 1.2 mg/dL when previously normal, or and an increase in serum creatinine by ≥0.3 mg/dL within 48 h or ≥1.5 times from baseline known to be normal within the prior 7 days, or urine volume is less than <0.5 mL/kg/h in the last six h.
- Acute hepatic failure was defined as a significant increase in serum transaminase levels accompanied by jaundice, coagulation disorder (INR of ≥ 1.5), and encephalopathy in a patient without cirrhosis or preexisting liver disease in the last seven days.
- Respiratory failure was defined as acute respiratory distress with PaO_2_ < 60 mmHg and/or PaCO_2_ > 45 mmHg in arterial blood gas analysis in room air.

Patients were excluded from the study based on the following criteria: patients who had an uncertain diagnosis of acute poisoning, who transferred to an external institution and whose data cannot be accessed, who already had organ failure(s) before index admission due to poisoning, who did not develop shock or any signs of organ failure during the emergency room follow-up, and who did not give written consent to participate in the study.

In addition to the demographic characteristics, the type of drug or substance they took, the way they were taken, and the dose were recorded. During the first application and follow-up of the patients, complete blood counts, biochemical tests, shock parameters, blood gas, and coagulation tests were studied. Patients were evaluated at the time of admission using the organic phosphate poisoning (POP) severity scale. POP is a scale designed to determine the prognosis of patients who present to the ED with organic phosphate poisoning. It is intended for the detection and severity of the toxidrome. However, it includes important parameters for all poisoning patients such as pupil diameter, respiratory rate, pulse rate, seizure, and consciousness [9]. Thus, the study planned to investigate whether POP can be used in other acute poisoning patients and whether other additional parameters are needed. The time of development of shock or acute organ failure, the type of acute organ failure, the treatment of poisoning, the type of antidotes used and the type of extracorporeal methods were recorded in detail, and the outcome of the patients was reported.

The conformity of the variables to the normal distribution was examined using histogram graphics and the Kolmogorov-Smirnov test. Mean, standard deviation and median values were used when presenting descriptive analyses. The Mann-Whitney U test was used when evaluating nonnormally distributed (nonparametric) groups. Fisher’s exact tests were used when making comparisons in categorical cells. Cases with a p-value below 0.05 were considered statistically significant results. The power of the study was calculated as 99%. All statistical analyses were carried out using SPSS (v. 23, IBM).

## 3. Results

A total of 89 patients were included. Seventy-two (80.9%) of the patients were male and 17 (19.1%) were female. The mean age of the patients was 49.1 years old (range: 19–90). While 63 (70.8%) patients were accidentally poisoned, 26 (29.2%) patients took the poison with the aim of suicide. Fifty-one patients (57.3%) were exposed to methanol, 12 patients (13.4%) to cardiovascular agents, six patients (6.7%) to aluminum phosphide, four patients (4.5%) to carbon monoxide, and four patients (4.5%) to mushroom.

The average length of hospitalization days was found to be 6.29 (range: 1–50) days. Unfortunately, 30 (33.7%) of the 89 patients with shock and acute organ failure died during hospitalization. The hospitalization days of all aluminum phosphide poisoning patients with shock and acute organ failure did not exceed 2 days.

Six (50%) of the 12 patients who took drugs from the cardiovascular group were given intravenous (IV) lipid emulsion therapy. One (3.3%) of the patients died despite all treatments.

Fifteen (29.4%) of the 51 patients diagnosed with methanol poisoning died. While fomepizole was used in 17 (19.1%) patients, ethyl alcohol was given to 36 (40.4%) and seven (7.9%) patients were given ethyl alcohol first and then fomepizole. While 45 (88.2%) of the patients were treated with hemodialysis, continuous renal replacement therapy (CRRT) was applied to seven (13.7%) patients. While the extracorporeal extraction method was applied to 12 of the 15 deceased patients, three patients died without receiving this treatment due to deep shock.

The agents responsible for poisoning and mortality in the study group are listed in Table 1 and Table 2, respectively. Methanol poisoning was the most common reason for both poisoning and death. Cardiovascular drugs were in the second leading cause of poisoning, while aluminum phosphide was the second highest cause of death.

The patients with a high POP score had a longer hospitalization period and higher mortality rate (p = 0.001) (Table 3).

The patients with hypotension detected at the time of admission had a higher mortality rate (p = 0.031). Intoxication patients who developed respiratory failure or coma or had tachypnea had a higher mortality rate than other patients (p = 0.001) (Table 4)

The mortality rate of poisoning patients who developed shock at the time of admission or in the first h was significantly higher than that of the patients who did not develop shock at the first h (p = 0.001)

The discharge rates of patients who received extracorporeal treatment were higher than those who did not receive this treatment (p = 0.001) (Table 5).

High WBC, creatine, lactate levels, base excess, and low bicarbonate and blood pH were associated with mortality (Table 6).

When the laboratory results of the patients included in the study were compared with the POP score, it was determined that high WBC, creatine, lactate levels, base excess, potassium and anion gap, and low blood pH and bicarbonate value were associated with the severity of poisoning (Table 7).

## 4. Discussion

Considering the previous studies, mortal poisoning continues to be an important cause of death in the young adult group [10,11]. When the studies with all poisoning patients are evaluated, it is seen that the female gender dominance is at the forefront, while when the mortal poisonings are examined, it is reported that the male gender is more dominant. Since the patient group in our study consisted of critical poisoning patients, it is seen that male gender dominance is in the foreground in accordance with the literature [12].

Methanol poisoning cases occurred in 57.3% of the patients. Due to the social and traditional characteristics of Turkey, the prevalence of the male gender is considered a natural consequence, since alcohol use is much higher among the male gender.

Mortality rates reported due to aluminum phosphide poisoning are quite high [13]. Ekinci et al. reported in their study that mortality rates after aluminum phosphide poisoning were between 40%–80% in the literature [14]. Among the cases of aluminum phosphide poisoning, only those with shock and acute organ failure were included in our study. Unfortunately, after this stage, despite all intensive care support, six of our patients with aluminum phosphide poisoning died. If shock and acute organ failure develop in high-dose aluminum phosphide intakes, the possibility of rescue is still very low despite various extracorporeal treatment methods.

Toxic alcohols still cause serious poisoning that can lead to death and permanent blindness all over the world. Considering the antidotes, having a higher affinity for alcohol dehydrogenase enzyme than ethyl alcohol, having fewer side effects, not needing blood level monitoring, and not affecting the blood anion gap and osmolal gap measurements are the advantageous sides of fomepizole. However, fomepizole can be quite expensive and difficult to obtain compared to ethyl alcohol. When the case series were examined, the mortality rate was 17% in patients who received fomepizole, while it was 22% in patients who received ethyl alcohol [15,16]. In our study, 36 patients were treated with ethyl alcohol, 17 patients with fomepizole, and seven patients were treated with ethyl alcohol first and then fomepizole together. Although fomepizole is a costly agent and hard to supply, it is nonetheless preferred in the first stage for some patients due to the side effects of ethyl alcohol (hypoglycemia, liver toxicity, etc.).

When poisoning patients who developed shock in the ED are examined, it is seen that most of them are poisoned due to cardiovascular drugs. In patients with shock secondary to cardiovascular drug intake, the treatment starts with fluid resuscitation by evaluating the volume status, but most of the shock in these patients is resistant to this treatment. Therefore, it may require an antidote, intensive vasopressor therapy, and lipid emulsion therapy. Extracorporeal treatment methods, such as pacemakers or intra-aortic balloon pumps, may also be required in these patients. A study conducted by Sebe reported that 80% of 15 patients (with shock) benefited from intravenous lipid therapy, but 20% continued to have resistant shock [17]. In our study, six of the 12 patients required lipid emulsion treatment; five of these patients responded to the treatment, and one patient died despite all medical treatment. Lipid emulsion therapy should definitely be considered in patients with resistant shock with cardiovascular agent intoxications. There is no clearly reported dosage of lipid therapy. Rapid infusions cause serious side effects in surviving patients. In addition to reported side effects, such as severe chest pain, shortness of breath, and problems such as lipid-related obstructions in the devices, difficulty in obtaining measurements due to intense and sudden lipid administration has been reported in extracorporeal treatments [18].

When mortality markers were evaluated in patients with acute critical poisoning, Eizadi-Mood et al. reported that the major differences between patients who died and those who were discharged were intubating the patients, being connected to a mechanical ventilator, and the development of aspiration pneumonia [19]. In our study, it was determined that intoxicated patients who were intubated or had tachypnea when they were applied to the ED had a higher mortality rate than that patients with normal respiratory rates, and there were statistically significant differences between them. In a study by Lee et al., the mortality rate of hypotensive patients was found to be significantly higher than that of the normotensive and hypertensive groups [20]. Since patients with shock and/or acute organ failure were included in our study when the time of development of shock was evaluated, it was found that the mortality of intoxication patients who presented to the ED or who developed shock in the first h was significantly higher than that of patients who did not develop shock in the first h (p = 0.001). For this reason, more dynamic follow-up of patients with hypotension in the early period and testing of advanced invasive extraction methods before the hypotension becomes too deep should be considered.

There are case reports and series showing that extracorporeal treatment methods can be used successfully in poisoning patients when the appropriate method is chosen at the appropriate time. In a multicenter study by Bouchard looking at the extracorporeal methods applied to acute poisoning patients, the cost and ease of transportation and the type of method applied were evaluated; intermittent hemodialysis (IHD) was mostly used (96.9%), followed by total plasma exchange (TPE; 68.3%), and continuous renal replacement therapy (CRRT). Bouchard reported that the use of peritoneal dialysis (PD; 44.8%) and hemoperfusion (HP; 30.9%) followed, and liver support devices (LSD, 14.7%) were used in patients who needed them. Considering the time to reach extracorporeal treatment in the study, it was seen that IHD, CRRT, and HP were the methods with the shortest time to reach treatment (median = 60 min) [21].

In our study, it was determined that the most easily accessible method was intermittent hemodialysis, followed by CRRT and total plasma exchange. When the relationship between the use of the extracorporeal method and survival rates was evaluated in the patients in our study, it was seen that hemodialysis, one of the extracorporeal treatment methods applied, increased the survival rates of the patients. It was determined that plasmapheresis was applied to four of the other patients in our study and erythropheresis was applied to four patients. It was determined that one of the patients who underwent plasmapheresis had opiate poisoning, two had mushroom poisoning, and one had paracetamol poisoning. Erythrocyte apheresis was applied to four carbon monoxide poisoning patients who had severe carbon monoxide poisoning and could not receive hyperbaric oxygen therapy due to respiratory failure due to mechanical ventilation. Three of four patients were successfully treated.

Undoubtedly, laboratory examinations are one of the most important parameters used when evaluating poisoned patients. Tang et al. reported that high lactate levels, low lactate clearance at the 6th h, and low blood pH were significant parameters for poor prognosis in patients with acute organic phosphate poisoning [22]. In a study conducted by Moghaddam et al. on patients with methyl alcohol poisoning, it was determined that a GCS of 9 and below, a pH < 7, and admissions later than 24 h were poor prognosis criteria [23]. In our study, it was determined that high WBC, creatine, lactate levels, base excess, and low bicarbonate and pH were significantly associated with mortality. In addition, high WBC, lactate levels, creatine, anion gap and base excess, and low pH and bicarbonate levels were associated with the severity of poisoning when compared with the POP scale.

The POP scale was calculated in the ED for all patients included in our study, and it was found that the mortality of patients with high POP scores was statistically significantly higher.

## 5. Conclusion

Accidental or suicidal acute poisoning is still a public health problem that can cause serious mortality. In our region, there is a need for serious control over the sale and supply of methanol and aluminum phosphide agents. The POP score can be easily calculated in the ED at the time of the first admission in all poisoning patients, which may partially help us predict the prognosis. Emergency physicians may consider the development of hypotension, high lactate and base excess, and low bicarbonate and blood pH to be poor prognostic markers in the early period. Therefore, the question of whether these criteria indicating perfusion impairment can be included in the POP score is raised. In selected patients, it was predicted that the addition of extracorporeal methods to the treatment without delay may increase survival rates.

## 6. Limitations

The major limitation of our study was that it was not proved poisoning by laboratory tests, not being revealed toxic levels for all study patients. A limited number of poisons, drugs and chemicals are assayed in our center, and list of these is given in the tables above. This is a single-centered observational study, which may seem to be a limitation, but being conducted in a referral university hospital for poisoning patients serving a large region in the southern area of Turkey may reflect the characteristics of the entire region. Because the area where the hospital is located is surrounded by agricultural land, this limits the generalizability of the findings to similar rural areas. Another limitation of the study is the admission time of patients, which might affect the outcome of any poisoning. Since there may be a delay in the treatment for patients who were not diagnosed and/or properly managed due to the inabilities of the facility from which they were transferred, some of the patients might be seriously affected and might have worse clinical conditions. The number of methanol poisonings during the study was more common than normal, making it difficult to generalize the findings.

## Figures and Tables

**Table 1:** The agents responsible for the poisoning.

Measurements		Number of patients (n)	%
**Names of drugs and poisonous substances**	Cardiovascular drugs (calcium channel blockers and beta blockers)	12	13.4
	Paracetamol	2	2.2
	2,4 Dinitrophenol	1	1.1
	Colchicine	2	2.2
	Aluminium phosphide	6	6.7
	Methanol	51	57.3
	Organic phosphate	3	3.4
	Opiate	2	2.2
	Carbon monoxide	4	4.5
	Mushroom	4	4.5
	Snake bite	2	2.2
**Total**		89	100.0

**Table 2:** The agents responsible for mortality.

Measurements		Number of patients (n)	%
**Agents of poisoning**	Cardiovascular drugs (calcium channel blockers and beta blockers)	1	3.3
	Paracetamol	1	3.3
	2,4 Dinitrophenol	1	3.3
	Colchicine	1	3.3
	Aluminium phosphide	6	20.0
	Methanol	15	50.0
	Organic Phosphate	2	6.7
	Opiate	1	3.3
	Carbon monoxide	1	3.3
	Mushroom	1	3.3

**Table 3:** Comparison of POP scales and hospitalization durations and outcome of the patients.

POP scale	Hospitalization duration		p
	Mean ± standard deviation	Min-max	
Mild poisoning (0–3)	5.05 ± 8.27	1–50	0.070
Moderate poisoning (4–7)	8.86 ± 10.87	1–40	
**POP scale**	**Outcome**		**p**
	**Exitus n (%)**	**Discharged n (%)**	
Mild poisoning (0–3)	8 (26.7)	52 (88.1)	**0.001**
Moderate poisoning (4–7)	22 (73.3)	7 (11.9)	

**Table 4:** Comparison of the patients’ vital signs and ICU outcomes.

Measurements		n	Outcome		p
			Exitus n (%)	Discharged n (%)	
**Blood pressure (mmHg)**	Low (≤90)	58	25 (83.3)	33 (55.9)	**0.031**
	Normal (91–130)	28	5 (16.7)	23 (39.0)	
	High (≥131)	3	0 (0.0)	3 (5.1)	
**Respiratory rate (per min)**	Normal (12–20)	19	2 (6.7)	17 (28.8)	**0.001**
	High (>20)	46	12 (40.0)	34 (57.6)	
	Intubated	24	16 (53.3)	8 (13.6)	

**Table 5:** Evaluation of the patients according to applied extracorporeal treatment modalities.

Measurements		n	Outcome		p
			Exitus n (%)	Discharged n (%)	
Applied extracorporeal treatment methods	No intervention	25	8 (26.7)	17 (28.8)	**0.001**
	Hemodialysis	42	7 (23.3)	35 (59.3)	
	Hemodialysis and hemofiltration	8	8 (26.7)	0 (0.0)	
	Hemodialysis and plasmapheresis	1	1 (3.3)	0 (0.0)	
	Hemofiltration	6	4 (13.3)	2 (3.4)	
	Hemofiltration and plasmapheresis	1	0 (0.0)	1 (1.7)	
	Plasmapheresis	2	1 (3.3)	1 (1.7)	
	Erythropheresis	4	1 (3.3)	3 (5.1)	

**Table 6:** Laboratory evaluations of the patients.

Measurements	Outcome		p
	Exitus (n = 30) Median (IQR)	Discharged (n = 59) Median (IQR)	
**WBC (10** **^3^** **/μL)**	13 (10.4–19.8)	10.5 (7.8–14.2)	**0.022**
**Hb (g/dL)**	13.4 (10.6–15.2)	14.6 (12.5–15.9)	0.055
**Hct (%)**	43.4 (33.6–48.9)	42.2 (37.4–48.9)	0.725
**Plt (10** **^3^** **/μL)**	222.5 (131–298)	209 (181–289)	0.859
**Glucose (mg/dL)**	143 (106–196)	125 (105–170)	0.325
**Creatine (mg/dL)**	1.4 (1–1.8)	0.9 (0.7–1.1)	**0.001**
**BUN (mg/dL)**	12.5 (10–20)	13 (10–21)	0.761
**Sodium (mEq/L)**	139 (135–144)	137 (135–140)	0.080
**Potassium (mEq/L)**	4.2 (3.7–5.5)	4.2 (3.7–5.1)	0.581
**Direct bilirubin (mg/dL)**	0.3 (0.2–0.7)	0.2 (0.1–0.4)	**0.032**
**Indirect bilirubin (mg/dL)**	0.5 (0.4–0.7)	0.4 (0.3–0.8)	0.110
**LDH (U/L)**	344.5 (251–807)	185 (143–260)	**0.001**
**AST (U/L)**	91.5 (47–187)	34 (23–67)	**0.001**
**ALT (U/L)**	46 (22–88)	22 (17–47)	**0.021**
**Amylase (U/L)**	110.5 (62–243)	93 (70–153)	0.712
**Sedimentation (mm/h)**	6.5 (2–18)	2 (2–17)	0.583
**Lactate (mEq/L)**	3.7 (2.1–6.7)	1.7 (1.1–2.4)	**0.001**
**CRP (mg/L)**	1.5 (0.3–4.7)	1 (0.5–4)	0.969
**pH**	7.1 (6.8–7.28)	7.31 (7.2–7.35)	**0.001**
**Bicarbonate (mEq/L)**	10.4 (7.4–14.6)	17.4 (8.4–21.6)	**0.007**
**Base excess**	−20.4 [(−27) – (−10.3)]	−8.3 [(−17.2) – (−3.4)]	**0.001**
**Anion gap**	24 (17–35)	22 (16–33)	0.253

**Table 7:** POP scores.

Measurements	POP score*		*p*
	Mild poisoning (n = 59) Median (IQR)	Moderate poisoning (n = 30) Median (IQR)	
**WBC (10** **^3^** **/μL)**	9.8 (6.4–13.8)	14.9 (11.6–22.3)	**0.001**
**Hb (g/dL)**	14.1 (12.3–15.9)	13.4 (11.5–15.7)	0.316
**Hct (%)**	42.6 (37–48.6)	44 (36.3–49.7)	0.603
**Plt (10** **^3^** **/μL)**	209 (158–290)	219.5 (159–298)	0.624
**Glucose (mg/dL)**	125 (105–162)	146.5 (107–208)	0.161
**Creatine (mg/dL)**	0.9 (0.7–1.2)	1.3 (1–1.7)	**0.001**
**BUN (mg/dL)**	12 (9–19)	14.5 (11–21)	0.268
**Sodium (mEq/L)**	137 (135–140)	140 (135–144)	**0.038**
**Potassium (mEq/L)**	4.1 (3.6–4.8)	4.5 (4–5.7)	0.064
**Direct bilirubin (mg/dL)**	0.2 (0.1–0.4)	0.3 (0.1–0.5)	0.442
**Indirect bilirubin (mg/dL)**	0.4 (0.3–0.9)	0.5 (0.2–0.6)	0.526
**LDH (U/L)**	192 (141–321)	279.5 (201–570)	**0.005**
**AST (U/L)**	39 (23–80)	60 (39–134)	**0.040**
**ALT (U/L)**	25 (17–49)	33 (18–62)	0.414
**Amylase (U/L)**	92 (65–135)	151 (81–309)	**0.012**
**Sedimentation (mm/h)**	2 (2–12)	14 (2–28)	**0.001**
**Lactate (mEq/L)**	1.7 (1.1–2.4)	3.4 (2.1–6)	**0.001**
**CRP (mg/L)**	1 (0.4–2.3)	3.4 (1.4–12)	**0.001**
**pH**	7.31 (7.21–7.35)	7.07 (6.8–7.25)	**0.001**
**Bicarbonate (mEq/L)**	17 (9.3–21.2)	9.1 (6.4–18)	**0.013**
**Base excess**	−10.1[(−16.7) – (−4)]	−20.8 [(−27) – (−8.6)]	**0.001**
**Anion gap**	22 (17–32)	30 (19–39)	**0.048**

* 0–3: Mild poisoning; 4–7: Moderate poisoning
